# A Color Indicator Based on 3-(4,5-Dimethylthiazol-2-yl)-2,5-diphenyltetrazolium Bromide (MTT) and a Biodegradable Poly(ester amide) for Detecting Bacterial Contamination

**DOI:** 10.3390/ijms25126671

**Published:** 2024-06-18

**Authors:** María José Lovato, María del Carmen De Lama-Odría, Jordi Puiggalí, Luis J. del Valle, Lourdes Franco

**Affiliations:** 1 Departament d’Enginyeria Química, Universitat Politècnica de Catalunya, Escola d’Enginyeria de Barcelona Est-EEBE, c/Eduard Maristany 10-14, 08019 Barcelona, Spain; maria.jose.lovato@upc.edu (M.J.L.); maria.de.lama@upc.edu (M.d.C.D.L.-O.); jordi.puiggali@upc.edu (J.P.); luis.javier.del.valle@upc.edu (L.J.d.V.); 2 Center for Research in Nano-Engineering, Universitat Politècnica de Catalunya, Campus Sud, Edifici C’, c/Pasqual i Vila s/n, 08028 Barcelona, Spain

**Keywords:** color indicator, MTT, bacteria, biodegradable, poly(ester amide), electrospinning

## Abstract

Bacterial contamination is a hazard in many industries, including food, pharmaceuticals, and healthcare. The availability of a rapid and simple method for detecting this type of contamination in sterile areas enables immediate intervention to avoid or reduce detrimental effects. Among these methods, colorimetric indicators are becoming increasingly popular due to their affordability, ease of use, and quick visual interpretation of the signal. In this article, a bacterial contamination indicator system was designed by incorporating MTT (3-(4,5-dimethylthiazol-2-yl)-2,5-diphenyltetrazolium bromide) into an electrospun PADAS matrix, which is a biodegradable poly(ester amide) synthesized from L-alanine, 1,12-dodecanediol, and sebacic acid. Uniaxial stress testing, thermogravimetric analysis and scanning electron microscopy were used to examine the mechanical properties, thermal stability, and morphology of the mats, respectively. The capacity for bacterial detection was not only analyzed with agar and broth assays but also by replicating important environmental conditions. Among the MTT concentrations tested in this study (0.2%, 2%, and 5%), it was found that only with a 2% MTT content the designed system produced a color response visible to the naked eye with optimal intensity, a sensitivity limit of 10^4^ CFU/mL, and 86% cell viability, which showed the great potential for its use to detect bacterial contamination. In summary, by means of the process described in this work, it was possible to obtain a simple, low-cost and fast-response bacterial contamination indicator that can be used in mask filters, air filters, or protective clothing.

## 1. Introduction

Environmental bacterial contamination has become a global public concern, as the infections originated by these microorganisms remain an important cause of mortality worldwide. Bacterial contamination in areas that must be sterile, such as those in the pharmaceutical, healthcare, and food sectors, represents a constant and worrying threat.

In the pharmaceutical industry, microbes present in the final product or even in the production process can cause the degradation of various components of the formulations, from simple sugars to complex aromatic compounds that can undergo chemical alterations in the presence of a suitable organism. Some bacteria, such as *Staphylococcus aureus,* which has been identified as a contaminant in some pharmaceutical products, are primary pathogens that cause illness even when accidentally administered and pose a risk to the general public [1]. On the other hand, certain organisms are classified as opportunistic pathogens because they are normally harmless to healthy adults but become infectious when the host’s resistance mechanisms are altered. Different *Staphylococcus* strains and other *Pseudomonas*, *Enterobacteria*, and *Flavobacterium* species are included in this bacterial classification [2].

In the healthcare system, hospital-acquired infections are an issue for patient safety [3]. For instance, it has been established that certain bacteria, such as *Acinetobacter baumannii*, *Pseudomonas aeruginosa*, and *Stenotrophomonas maltophilia*, are antibiotic-resistant and linked to a high mortality rate, increased healthcare expenses, and poorer clinical outcomes. Furthermore, over the last two decades, the *Burkholderia cepacia complex* has been regarded as the most prevalent bacteria in intensive care units, as well as a nosocomial pathogen associated with a number of outbreaks in immunocompromised patients, including pneumonia, peritonitis, bloodstream and urinary tract infections [4,5].

In the food industry, the vast majority of safe food preparation practices depend on a clean manufacturing space to deliver safe, high-quality products [6]. Bacteria can cause food spoilage or food poisoning through the production of enterotoxins. In dairy-processing environments, *Enterobacter*, *Lactobacillus*, *Listeria*, *Micrococcus*, *Streptococcus*, *Bacillus*, *Staphylococcus,* and *Pseudomonas* are the most frequently found bacteria on food contact surfaces [7,8]. Biofilms in the food sector may include bacterial species that are known to be harmful to healthy people or may exclusively affect individuals with impaired immune systems. These pathogens can cause food-borne diseases (*B. cereus*, *S. aureus*), as well as sometimes gastroenteritis (*E. coli*, *S. enterica*) and systemic disease (*E. coli* O157:H7, *L. monocytogenes*) [8,9]. Some methods commonly used to detect bacterial contamination in food are those based on polymerase chain reaction (PCR) [10], fluorescence, in situ hybridization flow cytometry, oligonucleotide DNA microarray [11], optical biosensors [12], amperometric electrode systems [13], mass-based biosensors [14], or color indicator film based on pH changes [15].

Therefore, not only is the targeted use of antimicrobial agents necessary to reduce the incidence of bacterial contaminants, but it is important to have methods to early detect bacterial activity with comparable accuracy to conventional microbiological assays. Under this premise, researchers have made considerable effort to create simple color-changing methods for detecting bacterial contamination. They offer several advantages, including low cost, compact size, and the possibility of direct and real-time detection [16]. Their ability to function depends on the interaction of the receptor and the analyte, which could result in a color response visible to the naked eye according to different concentrations of bacteria. Among these approaches, electrospun nanofibers have attracted particular interest due to their various qualities, such as flexibility, good chemical, physical, and mechanical properties, large surface area, and porosity [17]. Properly functionalized nanofibers have found applications in biomedical industries, such as wound healing, drug delivery, scaffolds, and sensors [18]. Generally, direct incorporation is the method used to functionalize nanofibers by adding substances, such as nanoparticles, organic compounds, dyes, nanomaterials, and biomolecules, to the polymer solution before electrospinning [16,19]. Recently, Sun et al. designed a color indicator film to detect bacteria in food by incorporating bromothymol blue (BTB) into degradable poly-L-lactic acid (PLLA) and polyvinylpyrrolidone (PVP) using the electrospinning technique [15].

The great potential of the color indicator films for bacterial detection has led us to develop a new detection system using MTT (3-(4,5-dimethylthiazol-2-yl)-2,5-diphenyltetrazolium bromide) as the indicator molecule for its simplicity, low cost, and fast color signal. The MTT assay is a popular colorimetric test that evaluates the activity of the enzymes responsible for converting MTT, a yellow tetrazolium salt, into the colorful product formazan [20,21]. The electrospun matrix was made of PADAS, a poly(ester amide) synthesized from L-alanine, 1,12-dodecanediol, and sebacic acid [22]. This polymer combines the rigidity and superior thermal and mechanical qualities of polyamides with the biodegradability of polyesters [23]. PADAS is hydrolytically degradable, which occurs primarily via ester bonds. Moreover, the polymer can be enzymatically degraded, being papain the most effective enzyme reported so far [22]. The degradation of the polymer into innocuous products agrees with some of the Sustainable Development Goals (13th and 15th principles).

The novelty of this study is to take advantage of the color change of the MTT embedded in an electrospun biodegradable PADAS mat when it is colonized by bacteria. These nonwoven mats will provide high surface-to-volume ratios and tunable porosities of up to 90% [21,22] that will improve their performance as detectors based on a perceptible color variation of the material that will depend on the sensitivity and response time [23]. Filters for face masks, air systems, or protective clothing that would incorporate the MTT-loaded electrospun PADAS fibers developed in this work will be endowed with the ability to detect bacterial contamination, which could be a good indicator to identify when the protective article needs to be changed or cleaned [24].

## 2. Results and Discussion

### 2.1. Preparation of MTT/PADAS Mats

**Figure 1:**
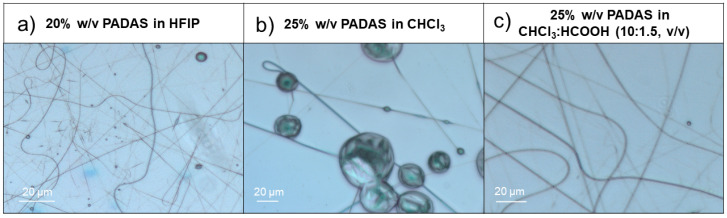
Optical images of PADAS electrospun fibers obtained with different solvents.

To produce a consistent nonwoven mat, several tests were performed to determine the ideal parameters of the electrospinning process. The variables that were optimized were solvent, polymer concentration, distance to the collector, flow rate, and voltage. First, 1,1,1,3,3,3-hexafluoro-2-propanol (HFIP) was utilized as a solvent because it was identified as the best option to obtain uniform fibers in previous research reported by del Valle et al. [25]. With HFIP, a 10% *w*/*v* solution of PADAS was tested, and as it was not viscous enough to form fibers, the concentration was increased to 20% *w*/*v*. With this higher concentration, uniform fibers were produced, but with some nanoparticles (electrospray), as shown in Figure 1a.

Alternatively, chloroform was similarly tested because it is a more economical solvent, less corrosive, and safer than HFIP. At 10% and 15% *w*/*v*, the solutions tested had a very low viscosity, which prevented the formation of fibers. It was necessary to increase the concentration up to 20% or 25% *w*/*v* (Figure 1b) to obtain fibers. However, even at that concentration, many beads were detected. To improve the quality of the fibers, formic acid was added in the ratio of 10:1.5 (*v*/*v*) to increase the conductivity of the solution and prevent bead formation. Under these conditions, PADAS concentrations of 20% and 25% *w*/*v* were tested. The latter concentration produced the best fibers, as shown in Figure 1c.

Once the best concentrations of solvent and PADAS (CHCl_3_:HCOOH 10:1.5 *v*/*v* and 25% *w*/*v*, respectively) were determined, the electrospinning operating variables were optimized. Collector distances from 10 to 25 cm, flow rates from 1 to 2 mL/h, and voltages from 15 to 30 kV were applied. The best fibers (Figure 1c) were obtained at a distance of 10 cm from the collector, with a flow rate of 1.2 mL/h and a voltage of 25 kV.

**Figure 2:**
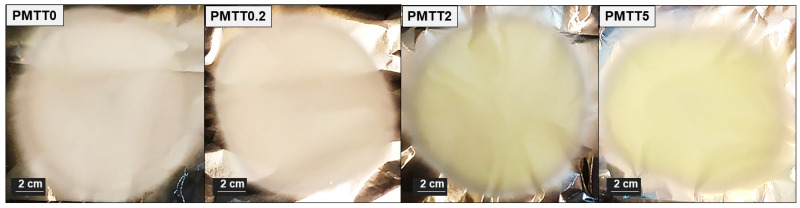
Photographs showing the appearance of MTT/PADAS mats.

For the development of MTT-loaded electrospun PADAS mats (MTT/PADAS mats), the concentrations of MTT used were 0.2%, 2%, and 5% (with respect to the weight of PADAS) in order to study how a relatively low, medium, and high amount of MTT influences the properties and the bacterial detection capacity of the mats. The terminology that was used hereafter to refer to MTT/PADAS mats is PMTT0, PMTT0.2, PMTT2, and PMTT5 for MTT concentrations of 0%, 0.2%, 2% and 5%, respectively. All the electrospun mats were obtained using the optimum electrospinning conditions summarized in Table 1. 1.15 mL of solution was used to produce a rounded mat approximately 12 cm in diameter and 0.1 mm thick. As expected, the intensity of the yellow color in the electrospun mats increases with MTT concentration (Figure 2).

### 2.2. Characterization of MTT/PADAS Mats

**Figure 3:**
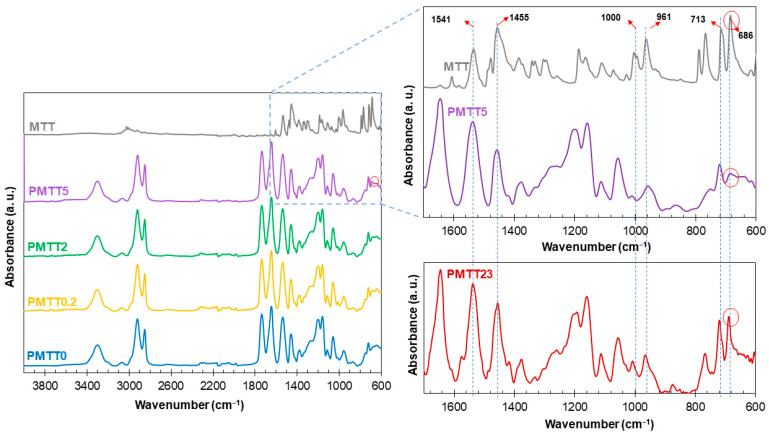
FTIR spectra of MTT and MTT/PADAS mats. For illustrative purposes, on the right side of the figure, an enlargement of the area of the spectra of interest to compare MTT and PMTT5 is presented. The dashed lines show the coincident bands between PADAS and MTT. The red circles show the 686 cm^−1^ peak, which is the only one confirming the presence of MTT in PMTT5 by this analysis technique. The spectrum of PMTT23 confirms that the 686 cm^−1^ peak becomes more intense at higher MTT concentrations.

In order to study the interaction of MTT with PADAS, the mats were characterized by FTIR (Figure 3). All spectra revealed the same bands typical of this poly(ester amide), as depicted in Figure S2a (Supplementary Information).

The specific bands corresponding to the MTT reagent are not observed in the mat spectra due to their low concentration and for having coincident bands with PADAS, such as 1541 cm^−1^ (C=C aromatic), which coincides with the amide II band of PADAS, or the 1455 cm^−1^ peak (C-C aromatic stretching, C=N stretching), which is at the same location as amide III of PADAS. Other characteristic bands of MTT as an aromatic compound are those located at 1000 cm^−1^ and 961 cm^−1^ (CH in-plane bending), 713 cm^−1^ (CH out-of-plane bending, C-N-C heterocyclic), and 686 cm^−1^ (aromatic ring deformation) [26]. This latter band (red circle) can be faintly visualized for the PMTT5 spectrum.

To illustrate that this band indicates the presence of MTT, a matrix was prepared with a higher concentration of MTT (23%, PMTT23). In the enlarged area of the IR spectra shown on the right side of the figure, the intensity evolution of this band as the MTT concentration increases can be clearly appreciated.

**Figure 4:**
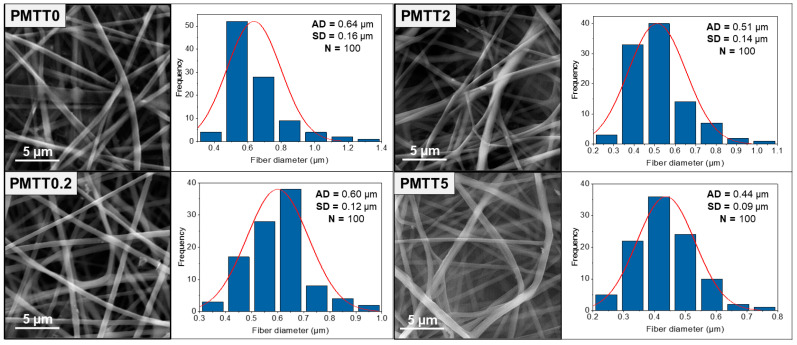
SEM micrographs of MTT/PADAS mats. AD stands for average diameter of fibers, SD for standard deviation, and N is the number of fibers measured.

To analyze in detail the morphological characteristics of MTT/PADAS mats, scanning electron microscopy (SEM) images were taken (Figure 4). As could be previously observed by optical microscopy (Figure 1c), all mats were homogeneous.

In general, the mats were composed of smooth and uniform bead-free fibers. The mean diameter was estimated by averaging the measurements of 100 fibers from each mat. As the MTT concentration increased, the fibers became thinner and had more disordered entanglements.

**Figure 5:**
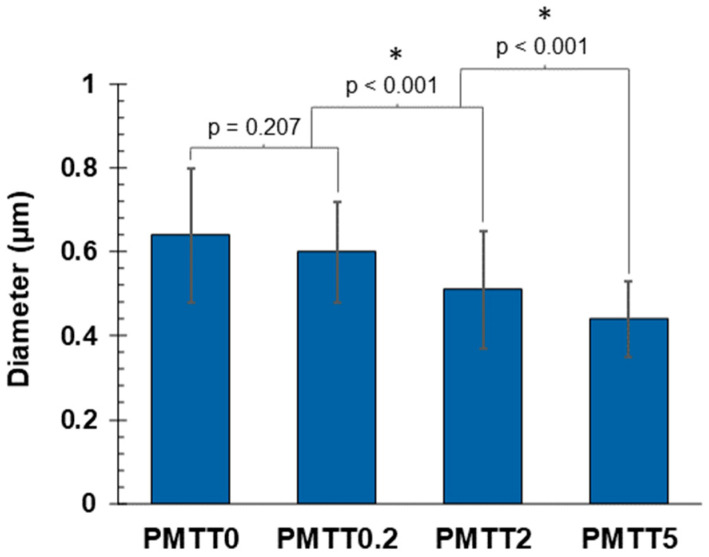
Mean fiber diameter of MTT/PADAS mats. Error bars represent the standard deviation. Data were analyzed by one-way ANOVA with a Tukey’s simultaneous test for mean differences (*p* < 0.05). * indicates a statistically significant difference between groups.

From the outcomes of ANOVA with a Tukey’s simultaneous test for mean differences (Table S1, Supplementary Information), it could be concluded that the diameters of the PMMT0 and PMTT0.2 fibers are statistically equal, which is logical because the amount of MTT added is minimal. For PMTT2 and PMTT5 fibers, there is a statistically significant difference, demonstrating that the fiber diameter decreases as the amount of MTT increases (Figure 5).

**Figure 6:**
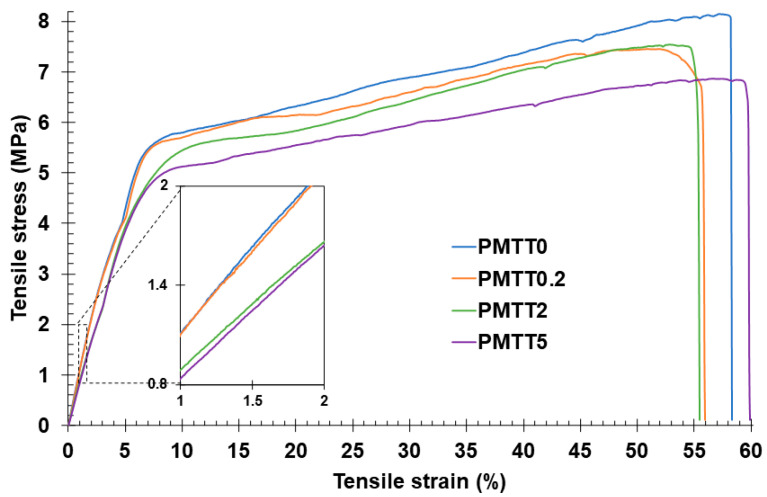
Stress–strain curves of MTT/PADAS mats.

Mechanical properties, including ultimate tensile strength and Young’s modulus, are crucial in assessing the ability of MTT/PADAS mats to resist tearing and rupture. To evaluate the influence of MTT incorporation, tensile assays were carried out. The recorded stress–strain curves and the corresponding mechanical parameter measurements are shown in Figure 6 and Table 2, respectively.

The stress–strain curves were very similar for all the MTT/PADAS mats tested and the mechanical properties barely differed. Although the PMTT5 presented a lower modulus, it is possible to conclude that the use of MTT at the concentrations employed in this investigation barely affects the mechanical properties of the material. The average Young’s modulus, ultimate tensile strength, and elongation were 80.6 MPa, 7.8 MPa, and 56%, respectively. Comparing these values with those reported for PADAS films obtained from the melt (Young’s modulus of 320 MPa, tensile strength of 18 MPa, and elongation at break of 10%) [27], it is clear that the electrospun PADAS becomes more elastic than the polymer film because of microstructural changes. This difference in the mechanical parameters could be explained by the fact that electrospinning generates ultrafine fibers with a high surface-to-volume ratio that tend to form porous structures.

The porous structure of fiber mats makes them more compliant and flexible. During the electrospinning process, polymer fibers are stretched uniaxially by an electric field, which can introduce defects and reduce the degree of alignment of the polymer chains [28]. This property results in a mat with a less organized and more amorphous structure than a film formed by melting the polymer.

Within the group of synthetically bonded nonwovens, polyamide fibers are one of the oldest used for a variety of applications due to their improved properties. Since the mats described in our work are made from a poly(ester amide), we consider that a comparison of their mechanical properties with those of polyamide would be appropriate. Data found in the bibliography related to Young’s modulus, ultimate tensile strength, and elongation of electrospun yarns/mats/membranes/scaffolds for nylon 6 (33.9 MPa, 7.2 MPa, and 120%, respectively) and nylon 66 (20.9 MPa, 6.5 MPa, and 140%, respectively) [29] fit rather well to the average values of the mats developed in this work. It can be observed that our MTT/PADAS mats have similar tensile strength values and less elasticity, which means that they can withstand approximately the same maximum tensile force before failure, despite being stiffer.

**Figure 7:**
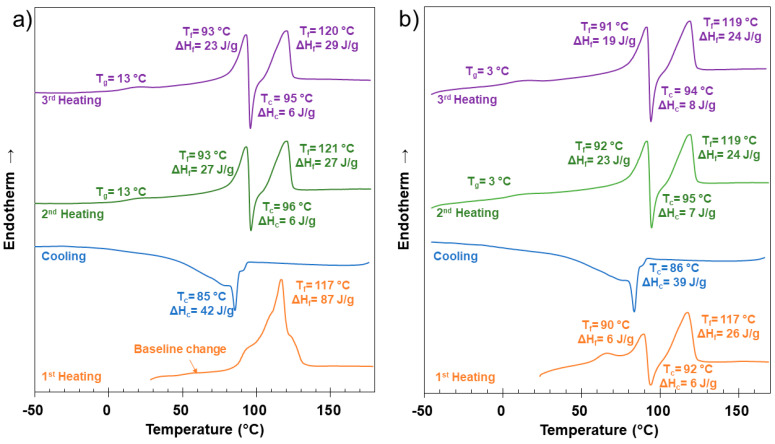
DSC curves corresponding to (**a**) as-synthesized PADAS and (**b**) PMTT2.

To corroborate the most amorphous character of samples made from electrospinning, the thermal behavior of PMTT2 was analyzed. As can be seen in Figure 7b, the melting enthalpy of both the melt-crystallized sample (2^nd^ heating run) and the quenched sample (3^rd^ heating run) was lower than that of the corresponding to PADAS as synthesized (Figure 7a). The lower degree of crystallinity is expected in an electrospun polymer, where the chains are less organized [28] could also explain a higher mobility of the polymeric chains and a higher free volume in the structure, which can lead to the reduction of Tg from 13 °C to 3 °C observed in PMTT2. A further explanation of DSC of PADAS can be found in Section S2 and Figure S3 (Supplementary Information).

Good interactions between the color indicator compound and the polymer matrix could be deduced from these results. The melting peak of PADAS shifted to a lower temperature when samples were loaded with MTT, which allows for inferring that the MTT was well mixed into the polymer phase. The lower enthalpy in the melting peak of PMTT2 indicated that MTT hindered the crystallization of PADAS and was incorporated into the polymer crystalline phase.

**Figure 8:**
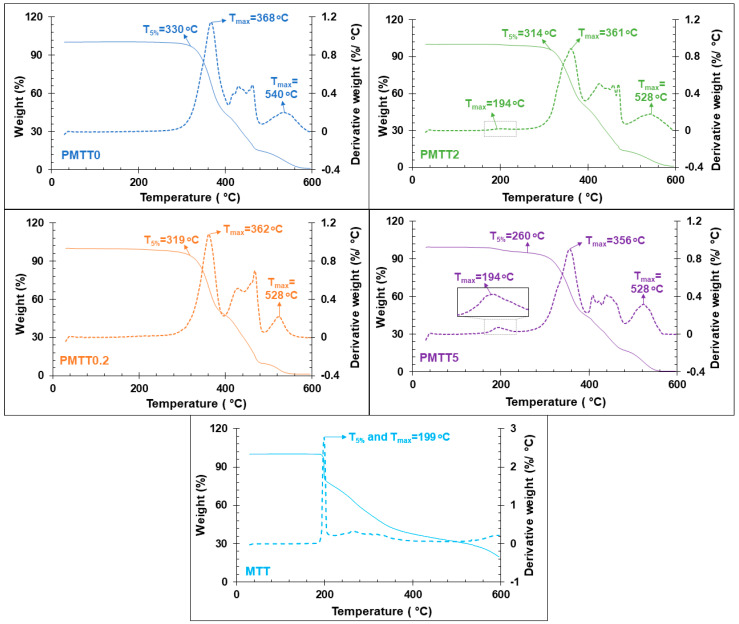
TGA (solid lines) and DTGA (dashed lines) curves of MTT/PADAS mats and MTT.

In addition to the mechanical properties, to evaluate the impact of MTT on the thermal stability of the mat, a thermogravimetric analysis (TGA) was conducted (Figure 8). Curves of the MTT reagent were also included for comparison purposes.

In all cases, a complex degradation process with more than three stages of decomposition was observed. For PMTT0, the first step appeared at 368 °C and the last at 540 °C. The arrows on the curves indicate the values of the initial degradation temperature measured at 5% weight loss (T_5%_) and the maximum decomposition temperature (T_max_). PMTT0, which begins to degrade at 330 °C, is the most thermally stable, whereas a small loss of stability is observed when the MTT content increases.

The evolution of the Tmax of the first degradation step is as follows: 368, 362, 361, and 356 °C for PMTT0, PMTT0.2, PMTT2, and PMTT5, correspondingly, whereas the Tmax of the last step is 540 °C for PMTT0 and 528 °C for all the MTT/PADAS mats. In contrast, for MTT, the curves showed only one main degradation step at 199 °C, and a 20% residue remained at 600 °C. Analyzing the DTGA curves of MTT/PADAS mats in detail, it could be observed that the main MTT degradation peak (Tmax = 194 °C) is only noticeable in PMTT2 and PMTT5 samples.

The surface wettability of the MTT-loaded electrospun PADAS mats was evaluated by measuring the contact angle of water droplets on the surface. The contact angles obtained were 128.27° ± 0.77, 127.11° ± 2.13, 126.18° ± 1.98, and 122.28° ± 1.48 for 0, 0.2, 2, and 5% of MTT content, respectively. Values showed a slight contact angle decrease when the MTT content increased. Generally, a contact angle > 90° indicates a hydrophobic surface [30]. The moisture content determined by the gravimetric method showed values ranging from 1.6% of PMTT5 to 1% of PMTT0, indicating that the material does not absorb humidity.

### 2.3. Bacterial Detection Experiments on MTT/PADAS Mats

The model organisms used for the bacterial detection experiments are strains of bacteria relatively safe to manipulate and culture in laboratory settings, such as *Escherichia coli* biofilm-negative CECT 101 (*E. coli*), *Escherichia coli* biofilm-positive (B+) CECT 434 (*E. coli* (B+)), *Ligilactobacillus salivarius* CECT 4063 (*L. salivarius*), *Streptococcus mutans* CECT 479 (*S. mutans*), and *Streptococcus sanguinis* CECT 480 (*S. sanguinis*). All of these bacteria may be potentially present in the oral microbiota. A further explanation of these bacteria can be found in Section S4 (Supplementary Information).

The McFarland method was used to estimate the cell concentration of the bacterial suspensions analyzed in this study. Figure S4 (Supplementary Information) shows the calibration curve obtained.

#### 2.3.1. Bacterial Detection Experiment in LB Broth

**Figure 9:**
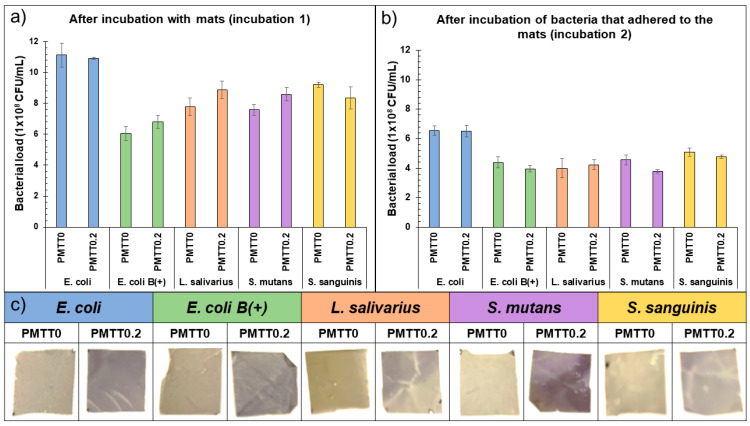
Bar plots of the bacteria count (**a**) after incubation in the presence of the electrospun mats and (**b**) after the incubation of the extracted bacteria that adhered to those samples. Error bars represent the standard deviation. (**c**) Appearance of the mats after the assay.

The prepared bacterial suspensions for the experiment had a cell density of 7.9 × 10^3^ CFU/mL, 6.7 × 10^3^ CFU/mL, 7.1 × 10^3^ CFU/mL, 3.5 × 10^3^ CFU/mL, and 6.3 × 10^3^ CFU/mL in the case of *E. coli*, *E. coli* B(+), *L. salivarius*, *S. mutans*, and *S. sanguinis*, respectively. Table 3 summarizes the bacterial count after 24 h of incubation of the listed bacteria suspensions in the presence of PMTT0 or PMTT0.2 mats (incubation 1). In addition, the bacteria density after 24 h of incubation of the recovered bacteria that adhered to the evaluated mats can be similarly consulted in this table (incubation 2). For comparative purposes, these results also depicted in Figure 9a and Figure 9b, respectively. From the results, it can be inferred that, regardless of the bacteria strain, there was a similar growth in the presence of MTT-free and MTT-loaded electrospun mats. Interestingly, the evaluated MTT concentration did not only negatively impact the bacterial growth but was also adequate to demonstrate their presence, as evidenced by the color change of the fibers from yellow to purple. This color variation was registered photographically after 24 h of incubation (Figure 9c).

It should be noted that the detachment of the bacteria that adhered to the samples after 24 h of incubation was performed to evaluate how these cells interact with the materials and to determine if the presence of the indicator molecule (MTT) could hinder that interaction. To attain this, the percentage of adhesion of each bacterial strain to the materials was calculated by dividing the bacterial count from incubation two by the calculated density after incubation one (values taken as the average between PMTT0 and PMTT0.2). Thus, the bacterial adhesion values were 59%, 65%, 49%, 52%, and 56% for *E. coli*, *E. coli* B(+), *L. salivarius*, *S. mutans*, and *S. sanguinis*, respectively. These results indicate a positive interaction, which, as previously pointed out, translates into a discernible visual response.

#### 2.3.2. Bacterial Detection Experiment in LB Agar

Since all bacteria used in the prior experiment were found to behave similarly and produce a discernible color change of the MTT/PADAS mats, only *E. coli* was selected for this experiment. The main objective of the test was to determine whether the MTT/PADAS mat samples could change color after a brief immersion of 5 s in the prepared bacterial suspensions. In order to provide the bacteria from the dipped samples with nutrients and humidity, the mats were placed on Petri dishes with LB agar. The obtained results served as a reference for the subsequent test, which aimed to study the potential of the MTT-loaded fibers to detect bacterial contamination when simulating in vivo conditions.

**Figure 10:**
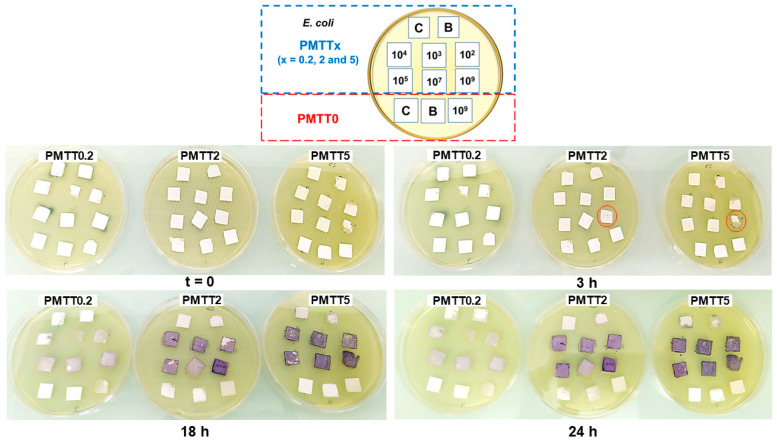
Comparison of PMTT0, PMTT0.2, PMTT2, and PMTT5 samples at different time points after being immersed in *E. coli* suspensions (cell density denoted by the 10^x^ values) or in sterile LB broth (blank group or B). C stands for control (samples that are not immersed in sterile LB broth or in the bacterial suspension).

The photographs of the Petri dishes at different time points are shown in Figure 10. At the beginning of the experiment, all the samples showed similar characteristics, despite the MTT concentrations. After 3 h, the samples of the PMTT2 and PMTT5 groups that were immersed in the *E. coli* suspensions (with a cell density of 10^9^ CFU/mL) started to show small purple dots, as highlighted by the red circles in the corresponding image.

After 18 h, for each tested MTT concentration, the samples that were submerged in the bacterial suspensions exhibited a total or partial color change to purple. Interestingly, the purple color intensity seemed directly proportional to the MTT concentration in the fibers. Moreover, the samples corresponding to the highest bacterial density (10^9^ CFU/mL) appeared more intensely colored.

In the images that correspond to the PMTT2 group, a purple color variation of the edges of the mats assigned as blank (“B” samples) could also be observed. A similar case was detected in the “B” and “C” samples of the PMTT5 group. These color shifts could be attributed to the bacteria’s migration across the LB agar surface and later colonization of the mats. As for the subtle alterations in color (to yellow) of the PMTT0 samples, this could be explained by a hydration process.

#### 2.3.3. Bacterial Detection Experiment Replicating Environmental Conditions

**Figure 11:**
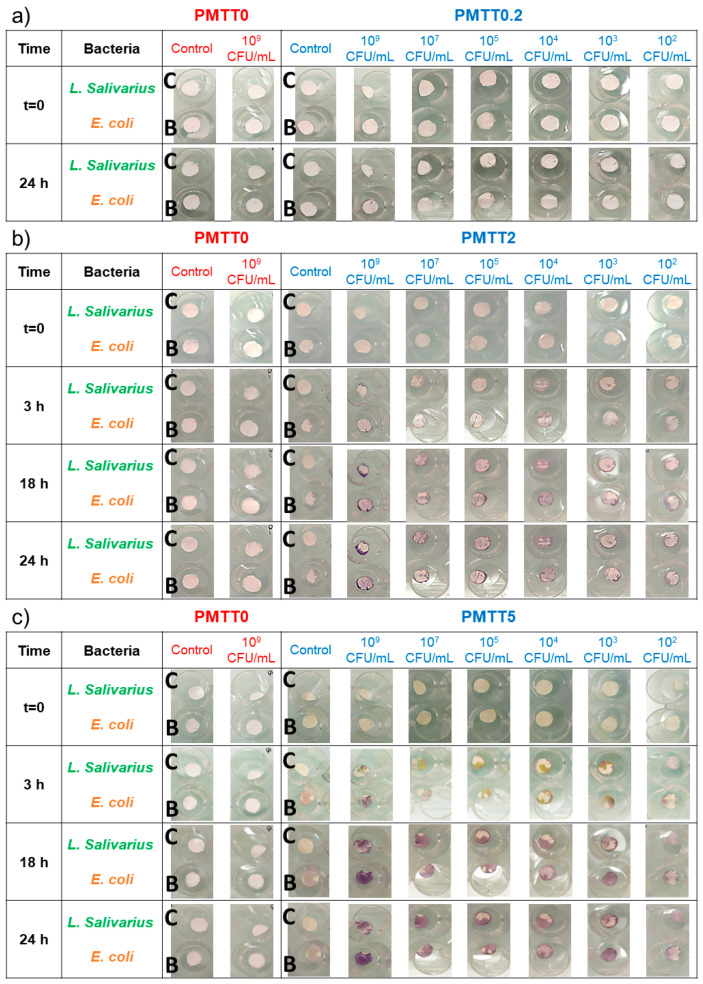
Photographic comparison of the bacterial detection limits of the MTT/PADAS mats when incubated under environmental conditions at different time points. PMTT0.2, PMTT2, and PMTT5 representative photographs are presented in (**a**–**c**), respectively. In all cases, “C” stands for control (samples that were not immersed in sterile LB broth or in the bacterial suspension), while B stands for blank (samples submerged in clean LB broth). For a better understanding, this figure was made by cutting the area of interest in the 24-well plates used to distribute the samples during the experiment. Figure S5 (Supplementary Information) shows the pictures without cuts.

This experiment aimed to evaluate what would happen if the MTT/PADAS mats were exposed to different bacterial concentrations and later incubated with no culture medium and with environmental humidity. These conditions would be most similar to those of the final application of mask filters, air filters, or protective clothing. Also, the test was designed to evaluate differences in bacterial contamination detection when exposed to either gram-negative or gram-positive bacteria. For this purpose, *E. coli* and *L. salivarius* were chosen as representative microorganisms, respectively. Furthermore, *E. coli* was used for the previous assays, while in the tests with LB broth (Section 2.3.1), *L. salivarius* showed the lightest purple tones, making these bacteria perfect for comparisons. A photographic comparison between the MTT-free and MTT-loaded PADAS mats at different time points after immersion in the bacterial suspensions can be seen in Figure 11.

In the case of MTT0.2 (Figure 11a), no color change was observed in the samples after 24 h (for this reason, only the first and last photographs of this group of samples are presented.), except for a few purple spots in the samples that were dipped in the highest bacterial density suspension. As a result, it was determined that this concentration of MTT would not be sufficient to be used in this type of bacterial contamination detector under environmental conditions.

For PMTT2 (Figure 11b), after 3 h, the first hints of purple pigmentation were seen in all samples, regardless of the bacteria classification or cell count. After 18 h, the purple intensity increased in each sample and remained constant until the end of the observation (24 h), with the most visible coloration occurring with bacterial counts from 10^4^ to 10^9^ CFU/mL. For the bacterial densities of 10^2^ and 10^3^ CFU/mL, there were visible purple stains at the edges of the samples, but the staining was not as intense and uniform as observed with higher cell numbers. Thus, the sensitivity limit of PMTT2 was established at 10^4^ CFU/mL.

PMTT5 exhibited behavior comparable to that described for the PMTT2 group. After 3 h, the sample that was submerged in the *E. coli* suspension of 10^9^ CFU/mL density was almost entirely colored. For samples corresponding to lower bacteria counts, the first hints of purple were just starting to develop. After 18 h, the purple hue in all samples increased and remained constant until the end of the observation (24 h). The sensitivity limit for this experimental group was finally determined to be 10^3^ CFU/mL.

Contrary to what was reported with previous assays, the lack of nutrients provided by a growth medium and the exposure of the samples to only environmental humidity were responsible for a lower purple color intensity and a heterogeneous pigmentation of the mats. As a result, it was not possible to detect differences in the detection capacity of the mats when exposed to gram-positive or gram-negative bacteria.

Regarding the bacterial cell number necessary to induce illness, the dose of an organism can differ significantly and is dependent on various factors, including the type of organisms present, the duration of exposure, the microbial load, the mode of administration, the patient’s age, nutritional status, and ability to fend off infection [31]. When exposure to bacteria occurs by the oral route, the dose required to cause adverse effects can be quite substantial; experiments with healthy individuals reveal that the infective dose of *Salmonella* or *E. coli* can be as high as 10^6^ to 10^7^ organisms, although positive results have also been observed with doses as low as 10^2^ to 10^3^. In relation to topical bacterial exposure, studies with healthy individuals revealed that an inoculum of up to 10^6^ *Staphylococcus aureus* may be sufficient to produce pus, but just 10^2^ may be enough if the skin is wounded [2]. Therefore, the sensitivity levels of 10^4^ CFU/mL or 10^3^ CFU/mL of environmental bacterial contamination obtained in this work can be considered of interest for further applications.

### 2.4. Cytotoxicity Evaluation of MTT/PADAS Mats

**Figure 12:**
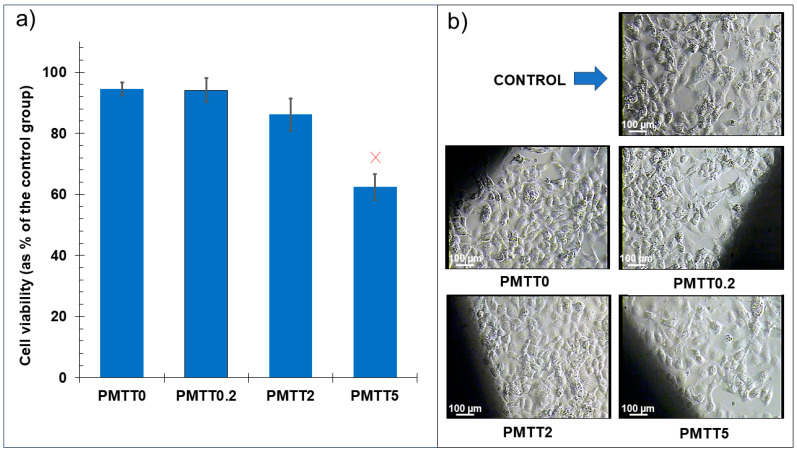
(**a**) Relative cell viability (results correspond to the mean, while the error bars indicate the standard deviation). (**b**) Representative micrographs of COS-1 cells grown adjacent to the tested MTT-free and MTT-loaded PADAS electrospun mats. A decrease in the cell number can be noticed in the PMTT5 group.

Samples of PMTT0, PMTT0.2, PMTT2, and PMTT5 were considered for an in vitro viability assay on COS-1 cells to assess the potential toxicity of any of the MTT/PADAS mats. As depicted in Figure 12a, the highest viability values were reported for the PMTT0 and PMTT0.2 groups, with a 94% value with respect to the control. Conversely, a decrease in viability could be observed with an increasing concentration of MTT. In this matter, 86% of viability was described in the PMTT2 group, while the cytotoxicity exhibited by the PMMT5 group reached 38% (62% of viability). The results of the viability assays could also be evidenced by optical microscopy. Representative micrographs of the growth of the COS-1 cells in the presence of the tested materials can be observed in Figure 12b. Due to the opacity of the fibers, the cells could not be visualized directly on the surface of the samples, so the presented micrographs correspond to the cells immediately adjacent to the mats (noticeable as the dark areas on each picture). Despite the lower value obtained with the PMTT2 mats, the viability percentage remains acceptable for the intended applications of the material. As for the PMTT5 fibers, the viability percentage is too low to be considered in future studies, so it was decided to mark them with a red X in Figure 12a.

Altogether, these results indicate that the PMTT0.2 and PMTT2 fibers could be estimated as potential materials. Nevertheless, further studies conceived to discard long-term toxicity are still needed.

## 3. Materials and Methods

### 3.1. Reagents

L-alanine, p-toluenesulfonic acid (p-TSA) monohydrate, 1,12-dodecanediol, toluene, sebacoyl dichloride, dichloromethane, diethyl ether, isopropanol, dimethyl sulfoxide (DMSO), and 1,1,1,3,3,3-hexafluoro-2-propanol (HFIP) (Sigma-Aldrich, Munich, Germany). 3-(4,5-dimethylthiazol-2-yl)-2,5-diphenyltetrazolium bromide (MTT) (Sigma-Aldrich, St. Louis, MO, USA). Sodium carbonate anhydrous, barium chloride dihydrate, and formic acid (Panreac, Barcelona, Spain). Sulfuric acid, chloroform stabilized with amylene, and Luria-Bertani (LB) Broth (Fisher Scientific, Roskilde, Denmark). Acetone (Honeywell, Düsseldorf, Germany), ethanol (Supelco, Munich, Germany), and Bacto Agar (Becton Dickinson, San Jose, CA, USA). All chemicals were analytical grade, and they were all used without further modification or purification.

### 3.2. Synthesis of PADAS

The synthesis process of the poly(ester amide) constituted by 1,12-dodecanediol, sebacic acid, and L-alanine (PADAS) was carried out in two steps following a method developed in previous studies [22]. The synthesis carried out in this work differs from the one previously reported in that dichloromethane was used in the interfacial polymerization instead of carbon tetrachloride. In Section S1 and Figure S1 (Supplementary Information), the process is explained in further detail.

### 3.3. Preparation of MTT/PADAS Mats

The solution electrospinning technique was used to prepare MTT/PADAS mats. The process was carried out under ambient conditions using a stationary collector located between 10 and 30 cm from the needle’s tip (21 G, internal diameter: 0.514 mm). Using a high-voltage power source (ES30-5W, Gamma High Voltage Research Inc. Company, Ormond Beach, FL, USA), voltages between 10 and 30 kV were applied. Solutions containing PADAS and MTT were delivered via a single infusion syringe pump (KDS100, KD Scientific Inc. Company, Holliston, MA, USA) to control the flow rate from 1 to 2 mL/h. Polymer solution flow rate, tip-collector distance, voltage, polymer concentration, and solvent were optimized to produce electrospun fibers.

### 3.4. Characterization of MTT/PADAS Mats

SEM observations were performed using scanning electron microscopy (Phenom XL Desktop SEM, Thermo Fisher Scientific Inc. Company, Waltham, MA, USA). Samples were displayed on a double-sided adhesive carbon disc and then carbon coated using a carbon evaporator (EM ACE600, Leica Microsystems Company, Wetzlar, Germany). Every sample was examined at a 10 kV accelerating voltage.

DSC of PMTT2 was performed with a differential scanning calorimeter (Q100 DSC, TA Instruments Company, Wilmington, DE, USA) equipped with a refrigerated cooling system (RCS) working in a controlled nitrogen atmosphere with a flow rate of 50 mL/min. The calibration of the equipment was performed with two experiments, with sapphire and indium. The sample (approximately 5 mg) was placed inside aluminum T-zero pans and subjected to a four-step protocol. First, a heating process was carried out at a rate of 10 °C/min. Next, a cooling process at a rate of 10 °C/min. Subsequently, a heating process was performed at a rate of 10 °C/min of the previously melted crystallized sample. Finally, a final heating run at 10 °C/min of the sample previously subjected to a very fast cooling process (50 °C/min). The protocol was carried out under dry nitrogen flow.

TGA of the samples was studied with a thermogravimetric analyzer (Q50, TA Instruments Company, Wilmington, DE, USA). TGA was performed under a nitrogen flow, with test temperatures ranging from −50 °C to 600 °C and a heating rate of 10 °C/min.

The mechanical properties of the samples were evaluated at room temperature by uniaxial elongation stress–strain assays performed with a material testing machine (Z2.5/TN1S, ZwickRoell S. L., Barcelona, Spain). Rectangular samples with dimensions of 4 × 0.4 cm^2^ and a thickness of around 0.10 mm were used for the measurements. After the samples were placed in the machine, they experienced continuous straining at a rate of 10 mm/min until breakage. The mechanical characteristics were obtained from the stress–strain curves recorded during the experiments.

The wettability of the mats was examined by contact angle measurements using a droplet shape analyzer (DSA25S, KRÜSS GmbH Company, Hamburg, Germany) equipped with a camera and Krüss Advance software (version 1.3.0.0). The sessile drop and ellipse methods were used to mathematically fit the drop shape. All measurements were performed at room temperature using a microsyringe to drop 2.0 µL of milli-Q water onto the surface of the MTT-loaded electrospun mats. Measurements were repeated five times on different surface locations, and the results were expressed as the average.

The moisture content of the mats was determined by the gravimetric method (loss on drying) described in ISO 15512:2019 [32]. In previously dried empty containers, MTT-loaded electrospun mats were placed and then dried to constant weight at 105 °C in an oven (UFB 400, Memmert GmbH Company, Büchenbach, Germany). The difference in weight of the samples was attributed to evaporated water and reported as moisture content.

### 3.5. Bacterial Detection Experiments on MTT/PADAS Mats

*Escherichia coli* biofilm-negative CECT 101, (*E. coli*), *Escherichia coli* biofilm-positive (B+) CECT 434 (*E. coli* (B+)), *Ligilactobacillus salivarius* CECT 4063 (*L. salivarius*), *Streptococcus mutans* CECT 479 (*S. mutans*), and *Streptococcus sanguinis* CECT 480 (*S. sanguinis*) were obtained from the Spanish Type Culture Collection (CECT).

#### 3.5.1. Bacterial Detection Experiment in LB Broth

**Figure 13:**
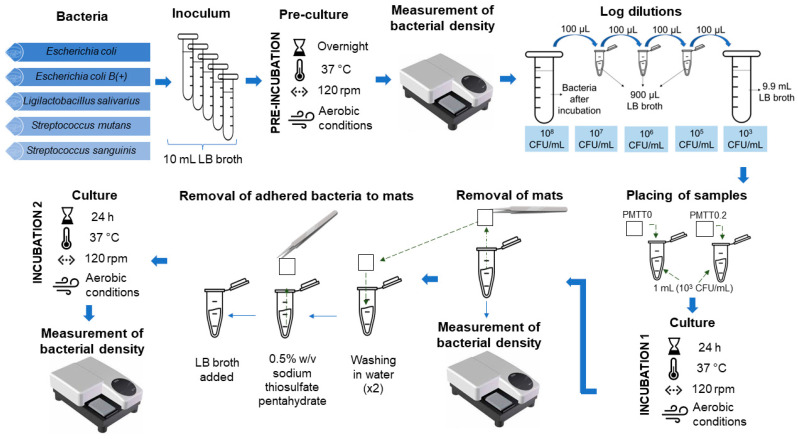
Flow chart of the bacterial detection experiment under incubation conditions with LB broth.

For this experiment, *E. coli*, *E. coli* (B+), *L. salivarius*, *S. mutans* and *S. sanguinis* were employed. Prior to the assays, bacteria were grown aerobically until reaching the exponential phase in LB broth. At this point, the obtained bacterial count was approximately 10^8^ CFU/mL. From these suspensions, successive log dilutions were prepared in fresh LB broth until a bacterial density of 10^3^ CFU/mL was achieved (Figure 13).

During the bacteria detection assays, PMTT0.2 mats of 1 cm^2^ were placed inside sterile Eppendorf tubes containing 1 mL of the 10^3^ CFU/mL bacteria suspensions, followed by a 24 h aerobic incubation at 37 °C and under constant agitation. PMTT0 mats with similar dimensions were similarly treated to be used as a control group. After incubation, the LB medium with grown bacteria was carefully collected from each tube, and the bacterial density was estimated by the McFarland standard. The mats were simultaneously subjected to extensive double washing in 1.5 mL of autoclaved milli-Q water. The washing medium was discarded, and the fibers were immediately covered with 0.5 mL of a 0.5% w/v aqueous solution of sodium thiosulfate pentahydrate, followed by a 30 min incubation process at room temperature with no agitation to extract the cells adhered to the samples. Next, 1 mL of fresh LB broth was poured to evaluate the adhered bacteria in each sample. The Eppendorf tubes were then incubated at 37 °C under continuous stirring for 24 h.

Next, 1 mL of fresh LB broth was added to evaluate the adhered bacteria in each sample. The treated mats were again incubated for 24 h under the already-described conditions. After this time, the microbial density of each tube was determined using the McFarland method. An overview of the explained protocol is presented in Figure 13.

#### 3.5.2. Bacterial Detection Experiment in LB Agar

**Figure 14:**
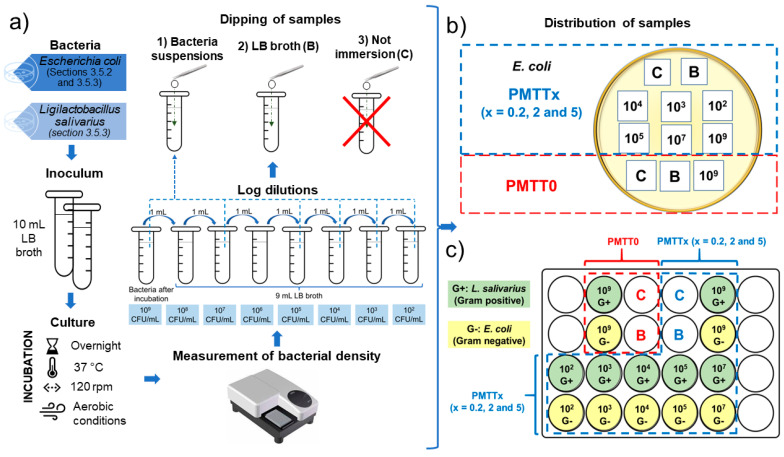
(**a**) Flow chart followed to dip the electrospun PADAS mats in bacterial suspensions or in sterile LB broth. (**b**) Distribution of the prepared PMTT0, PMTT0.2, PMTT2, and PMTT5 mats in the Petri dishes with LB agar. (**c**) Distribution of samples of PMTT0, PMTT0.2, PMTT2, and PMTT5 in 24-well plate. In (**b**,**c**), C stands for control (samples that were not immersed in sterile LB broth or in the bacterial suspension), while B stands for blank (samples submerged in clean LB broth). In all cases, 10× represents the bacterial cell density of the prepared suspension into which the samples were immersed.

*E. coli* was chosen for this experiment. Log dilutions in fresh LB broth were performed from a suspension of bacteria grown aerobically to exponential phase until cell densities of 10^9,^ 10^7^, 10^5^, 10^4^, 10^3^, and 10^2^ CFU/mL were achieved. For the assay, square mats of 1 cm^2^ were prepared from the MTT-loaded electrospun PADAS fibers. PMTT0 samples were similarly prepared to be used as a control. The samples were later sterilized for 15 min under UV light (310 nm) inside a type I safety cabinet. From this point, all samples were divided into three groups: (1) samples to be immersed for 5 s in the prepared *E. coli* suspensions, (2) the samples to be submerged into sterile LB broth (hereafter the blank group or B samples), and (3) the samples that will not be immersed in any medium (hereafter the control group or C samples) (Figure 14a).

Subsequently, the mats were transferred to Petri dishes with LB agar. The dishes were kept inside the laminar flow hood at room temperature for 24 h in order to avoid cross-contamination during the incubation (Figure 14b). To visualize the color change of the mats, photographs were taken at the following time points: 0 h, 3 h, 18 h, and 24 h.

#### 3.5.3. Bacterial Detection Experiment Replicating Environmental Conditions

For this experiment, gram-negative *E. coli* and gram-positive *L. salivarius* were considered. Bacterial suspensions with the same cell density as the ones selected for the experiments in Section 3.5.2 were made (Figure 14a). The sample preparation for this experiment differs from the above-mentioned protocol in that instead of squares, 8 mm diameter circular samples were cut from each of the MTT-loaded PADAS and PMTT0 electrospun mats. All the samples were sterilized as already described. After this process, the samples were divided into three different groups that were treated as described in Section 3.5.2 (Figure 14a). However, the treated samples were later transferred to a 24-well plate instead of Petri dishes (see Figure 14c for a reference of the sample distribution). During the incubation, the plates remained inside the laminar flow hood to avoid cross-contamination. Photographs were taken at the same time points considered in Section 3.5.2 to visualize the color change of the mats.

### 3.6. Cytotoxicity Evaluation of MTT/PADAS Mats

COS-1 cells (ATCC CRL-1650) were selected for this study. The protocol followed is based on the ISO 10993-5:2009 standard [33], which specifies the proper approach for evaluating the in vitro cytotoxicity of medical devices. MTT-loaded electrospun PADAS and PMTT0 mats were cut into square 1 cm^2^ pieces. These samples were transferred to a 24-well plate and sterilized for 15 min under UV light inside a type II safety cabinet. Later, an aliquot of DMEM high glucose medium containing 1 × 10^5^ cells was seeded in each well and incubated for 24 h (37 °C, 5% CO_2_). Cells directly seeded in empty wells were used as a growth control group. After incubation, an MTT assay was performed to assess the cell viability. Briefly, 50 µL of a 5 mg/mL MTT solution (prepared in PBS 1×) was added to each well, and the plate was incubated for 4 h under the already described conditions.

Afterward, the medium was removed, and the samples were washed twice with PBS. Subsequently, 1 mL of DMSO was added to each well, and the plate was gently shaken for 15 min, protected from light. To determine the absorbance at 570 nm, 100 uL from each well was transferred to a 96-well plate. Measurements were carried out in a microplate reader (Biochrom EZ Read 400, Fisher Scientific S.L. Company, Madrid, Spain). The experiments were performed in triplicate, and the results are presented as the mean ± SD. To calculate relative percentages, viability values were standardized with respect to the control.

## 4. Conclusions

In this paper, mats from MTT and PADAS, a biodegradable poly(ester amide), were successfully prepared by electrospinning technique. Good interactions between the color indicator compound and the polymer matrix could be inferred from infrared and thermal data.

The hydrophobic character of the surface (contact angle around 126°) and the mechanical properties (average Young’s modulus, ultimate tensile strength, and elongation of 80.6 MPa, 7.8 MPa, and 56%, respectively) of the electrospun mats remained practically unaltered with MTT loading.

It has been demonstrated that the incorporation of MTT in PADAS matrices allows the design of a system to detect bacterial contamination since the mats containing MTT had good color responsiveness. Specifically, a 2% MTT incorporation is sufficient to have a visual response with optimal intensity. In the bacterial detection test, the color indicator mat could show a sensitivity limit of 104 CFU/mL for the gram-positive and gram-negative bacteria tested and 86% cell viability.

In summary, through the process described in this work, it is possible to obtain a simple, low-cost and fast-response bacterial contamination indicator that can be used in mask filters, air filters, or protective clothing. Furthermore, it opens the possibility to develop detectors for specific viruses or bacteria, varying the active component incorporated into the matrix.

## Figures and Tables

**Table 1:** Optimum electrospinning conditions.

Parameter	Value
Internal needle diameter	0.514 mm (21 G)
Collector type	Flat surface
Solvent	CHCl_3_:HCOOH (10:1.5, *v*/*v*)
PADAS concentration	25% *w*/*v*
MTT concentration	0.2, 2, 5% *w*/*w*
Flow rate	1.2 mL/h
Voltage	25 kV
Distance (needle tip to collector)	10 cm

**Table 2:** Main mechanical parameters.

Parameter	PMTT0	PMTT0.2	PMTT2	PMTT5
Young’s Modulus (MPa) *	83.29 ± 4.31	82.12 ± 1.64	79.14 ± 2.60	77.68 ± 2.21
Ultimate Tensile Strength (MPa) *	8.17 ± 0.20	7.54 ± 0.37	7.32 ± 0.36	7.98 ± 0.79
Elongation (%) *	58.34 ± 6.00	53.76 ± 10.90	55.12 ± 6.08	54.96 ± 6.69

* Results are presented as the mean ± standard deviation (n = 5).

**Table 3:** Bacterial count (×10^8^ CFU/mL) of the LB medium incubated with the PMTT0 and PMTT0.2 mats and of the suspensions of the bacteria that adhered to those samples.

	Bacterial Density after Incubation with Mats (24 h) *		Cell Density of the Suspensions of Bacteria That Adhered to the Mats (24 h) *	
Bacteria	PMTT0	PMTT0.2	PMTT0	PMTT0.2
*E. coli*	11.14 ± 0.77	10.93 ± 0.08	6.56 ± 0.32	6.53 ± 0.40
*E. coli B(+)*	6.05 ± 0.45	6.80 ± 0.42	4.39 ± 0.39	3.96 ± 0.23
*L. salivarius*	7.79 ± 0.56	8.88 ± 0.57	3.99 ± 0.66	4.24 ± 0.35
*S. mutans*	7.59 ± 0.35	8.59 ± 0.44	4.56 ± 0.33	3.79 ± 0.12
*S. sanguinis*	9.21 ± 0.18	8.36 ± 0.73	5.09 ± 0.29	4.77 ± 0.11

* Results are presented as the mean ± standard deviation (n = 3).

## Data Availability

Data is contained within the article and Supplementary Materials.

## Supplementary Materials

The supporting information can be downloaded at: https://www.mdpi.com/article/10.3390/ijms25126671/s1. References [34,35,36,37,38,39,40,41,42,43,44,45,46,47,48,49,50,51,52] are cited in the supplementary materials.
