# Supplementary material for: A Color Indicator Based on 3-(4,5-Dimethylthiazol-2-yl)-2,5-diphenyltetrazolium Bromide (MTT) and a Biodegradable Poly(ester amide) for Detecting Bacterial Contamination
Source: Int J Mol Sci. 2024 Jun 18;25(12):6671. doi: 10.3390/ijms25126671 (PMC11204193; doi:10.3390/ijms25126671)
Supplement: Supplementary file 1 [file ijms-25-06671-s001.zip › ijms-3017884-supplementary.pdf]

A Color Indicator Based on 3-(4,5-dimethylthiazol-2-yl)-2,5-diphenyltetrazolium bromide (MTT) and a Biodegradable Poly(ester amide) for Detecting Bacterial Contamination

Supplementary Information

María José Lovato[1], María del Carmen De Lama-Odría[1], Jordi Puiggalí[1,2], Luis J. del Valle[1,2] and Lourdes Franco[1,2,*]

1 Departament d'Enginyeria Química, Universitat Politècnica de Catalunya, Escola d'Enginyeria de Barcelona Est-EEBE, c/Eduard Maristany 10-14, 08019 Barcelona, Spain

2 Center for Research in Nano-Engineering, Universitat Politècnica de Catalunya, Campus Sud, Edifici C', c/Pasqual i Vila s/n, E-08028 Barcelona, Spain

* Author to whom correspondence should be addressed.

S1. Synthesis of PADAS

The synthesis process of the poly(ester amide) constituted by 1,12-dodecanediol, sebacic acid, and L-alanine (PADAS) was carried out in two steps as shown in Figure S1 and following a method detailed in previous studies [1]. The first step consisted of the preparation of monomer 1. L-alanine (0.09 mol), p-TSA monohydrate (0.09 mol), and 1,12-dodecanediol (0.045 mol) were charged into a Dean-Stark apparatus in the presence of toluene (200 mL) as solvent. The reaction mixture was then refluxed overnight until no more water was distilled out. When the reaction was complete, the mixture was allowed to cool to room temperature, and a thick oil was separated from the toluene solution. After drying at vacuum, the recovered solid was recrystallized twice from isopropanol. The purified white powder was monomer 1.

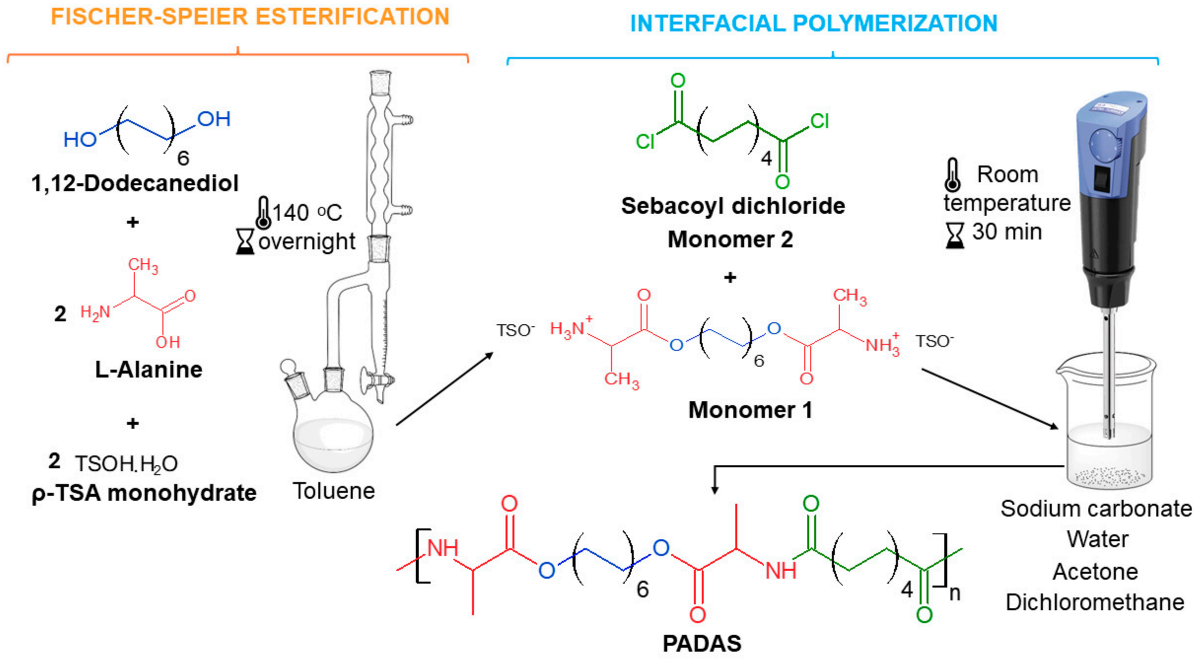

Figure S1. Synthesis of PADAS (**P**oly(ester amide) derived from L-**A**lanine, 1,12-**D**odecanediol, L-**A**lanine, and **S**ebacic acid), as previously reported in [1]

In a second step, the interfacial polymerization at room temperature of monomer 1 and sebacoyl dichloride (monomer 2) was conducted. For this purpose, a solution of monomer 1 (0.015 mol) and sodium carbonate (0.03 mol) was prepared in an approximately 15:1 water:acetone mixture (155:10 mL). Later, this solution was added to a solution of sebacoyl dichloride (0.015 mol) in dichloromethane (75 mL). The 30 min reaction was conducted under vigorous stirring. The immiscible polymer was filtered from the reaction media and successively washed with water, ethanol, and diethyl ether before drying under vacuum at 60 °C [23]. L-alanine was used to obtain enantiomeric pure L-PADAS.

S2. Characterization of PADAS

FTIR absorption spectra of monomer 1 and PADAS were taken using a Jasco FTIR 4100 Fourier transform instrument equipped with an attenuated total reflection (ATR) top plate and 4 cm^{-1} of resolution. The measurement range was 4000-600 cm^{-1}. ^{1}H-NMR spectra of monomer 1 and PADAS were obtained using a Bruker Ascend 400 MHz NMR spectrometer. As internal standard, tetramethylsilane (TMS) was used to calibrate chemical shifts. Deuterated trifluoracetic acid and deuterated chloroform were used as solvents for monomer 1 and PADAS, respectively.

GPC was performed with a Shimadzu LC-8A liquid chromatograph equipped with a Shimadzu RID-10A refractive index detector. The PADAS sample was dissolved and eluted in HFIP containing sodium trifluoroacetate (0.05 M) through an Agilent PL HFIP gel column. Agilent GPC/SEC polymethyl methacrylate standards were used for the calibration curve.

DSC was made as explained in section 3.4.

To verify the polymer synthesis, PADAS was characterized by means of Fourier-transform infrared (FTIR) and proton nuclear magnetic resonance (^{1}H-NMR) spectroscopies (Figure S2).

Specific absorption bands of poly(ester amide)s can be observed: amide A (3306 cm^{-1}, N-H stretching), amide B (3067 cm^{-1}, N-H stretching), amide I (1644 cm^{-1}, C=O stretching + C-N bending), amide II (1541 cm^{-1}, C-N stretching, N-H bending), amide III (1455 cm^{-1}, C-N stretching, N-H bending), amide V (678 cm^{-1}, out of the plane motions), ester (1733 cm^{-1}, C=O stretching; 1204 cm^{-1} and 1058 cm^{-1}, C-O-C stretching) and methylene (2920 cm^{-1} for C-H asymmetric stretching, 2851 cm^{-1} for C-H symmetric stretching and 719 cm^{-1} for C-H bending). The assignment of the signals for the different hydrogen atoms of the constitutive repeat unit of PADAS in the ^{1}H-NMR spectrum is included in Figure S2b.

Size exclusion chromatography (GPC) (profiles not shown) demonstrated that molecular weight values were suitable for film and fiber formation. The obtained weight average molecular weight (Mw) of 20,000 g/mol, number average molecular weight (Mn) of 8,300 g/mol, and polydispersity index (PDI) of 2.71 were consistent with those estimated in previous studies [1-3].

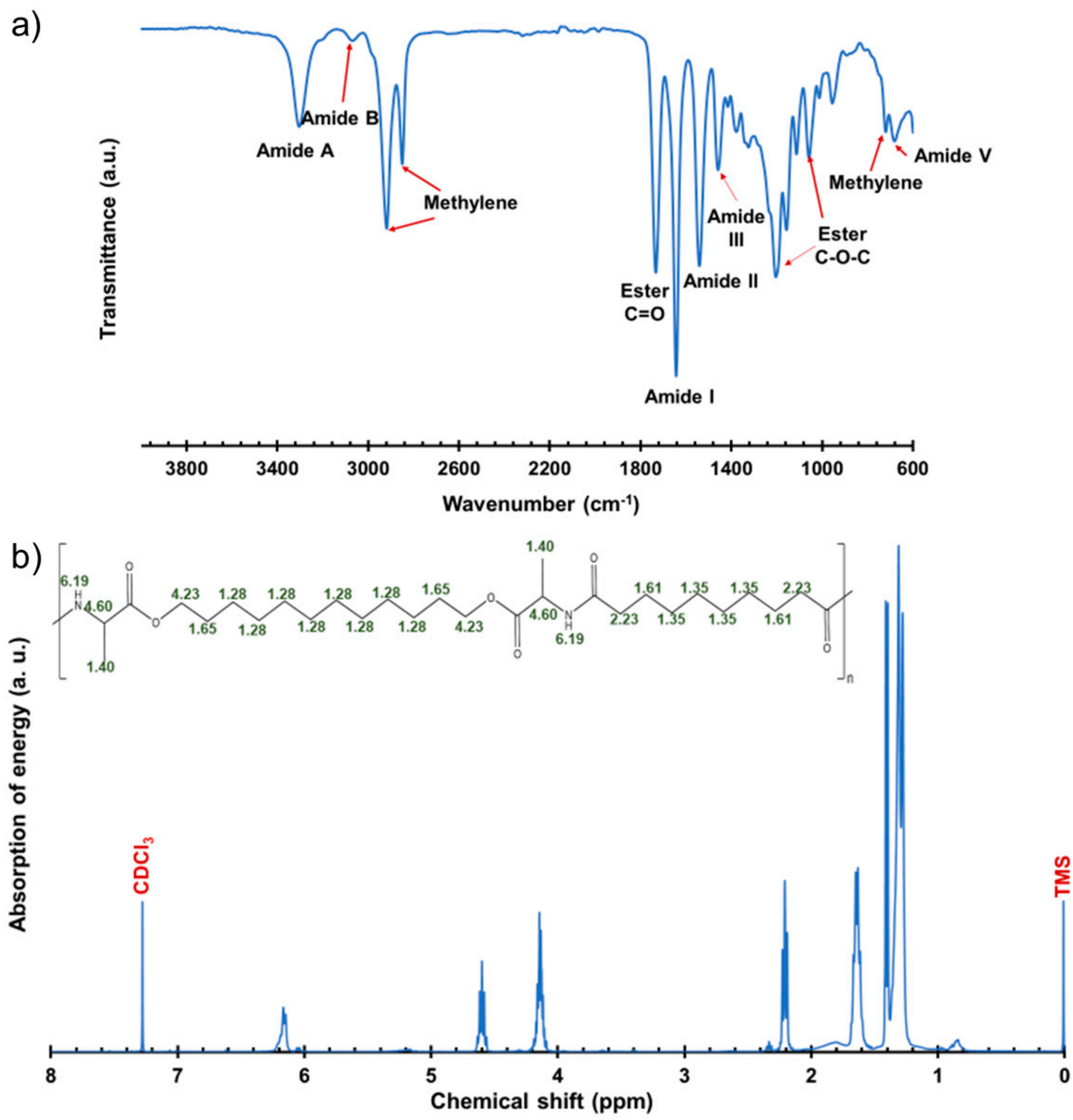

Figure S2. a) FTIR spectrum with functional groups labelled and b) ^{1}H-NMR spectrum with the corresponding chemical shifts of H atoms in the formula of as-synthesized PADAS

Figure S3 shows the differential scanning calorimetry (DSC) heating and cooling runs at a rate of 10 °C/min performed for PADAS as synthesized. The high enthalpy measured in the first heating indicated that samples crystallized from solution were highly crystalline. In the first heating and cooling cycle, complex melting (Tf = 117 °C) and crystallization (Tc = 85 °C) peaks were observed. In the second and third heating runs, the "complex peak" is transformed into double melting peaks (93 °C and 121 °C) with a hot crystallization peak (Tc = 96 °C) in between. This behavior is a typical feature of polymers and indicates a re-crystallization process where thin lamellae formed during the cooling run become thicker. The quenched sample also presented a hot crystallization peak at 95 °C between the two indicated melting peaks (93 °C and 120 °C).

As reported in earlier studies for L-PADAS and D,L-PADAS [1,4], a baseline change can be observed in the first heating around 60 °C (as shown with an arrow in Figure S3, a temperature close to the T_g of polyamides, suggesting that the amorphous part of the sample recovered from polycondensation is mostly constituted by peptide bonds.

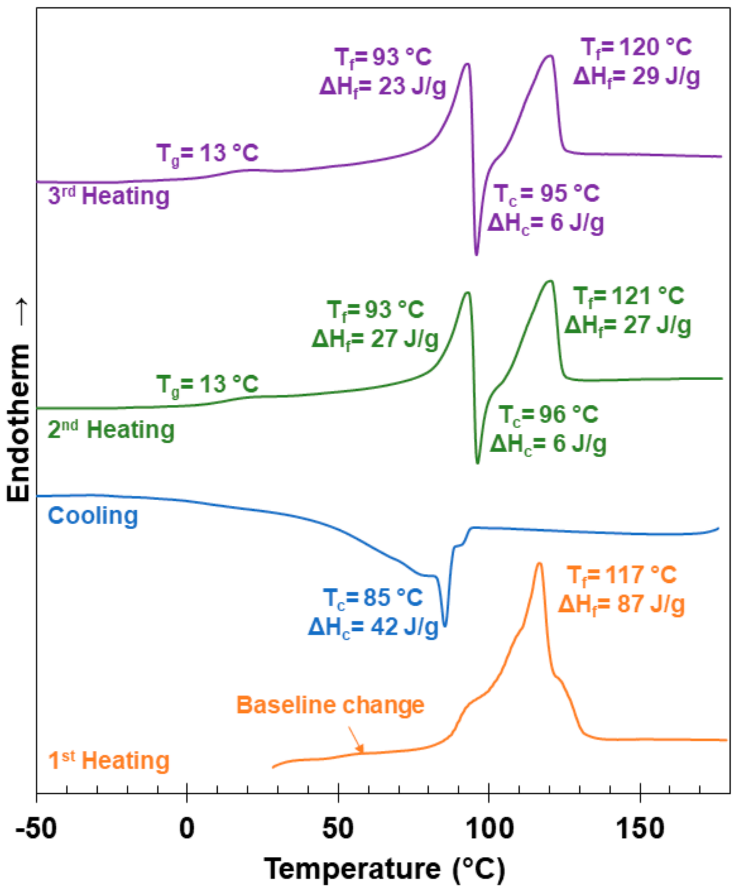

Figure S3. DSC curves corresponding to as-synthesized PADAS

On the contrary, a value of 13 °C was observed for melt crystallized and quenched samples indicating that in this case that amorphous part has an equilibrated ratio between ester and amide segments. The crystallinity ($W = \Delta H_f / \Delta H_f^{eq}$) of the sample could be evaluated by using the heat of fusion for a 100% crystalline material (88.4 KJ/mol) determined from the reported group contributions of ester, amide, $CH\text{-}CH_3$ and methylene groups (-2.5, 2.0, 4.7 and 4.0 kJ/mol, respectively) [5], and the observed heats of fusion ΔH_f. Thus, crystallinities of 27.4% and 26.2% were estimated for melt-crystallized and quenched-crystallized samples, respectively, values quite similar to those observed by Paredes *et al.* (25.3% and 21.1%) [1].

S3. Fiber diameter

The outcomes of the ANOVA with a Tukey's simultaneous test for mean differences are presented in Table S1. Levels 1, 2, 3, and 4 represent PMTT0, PMTT0.2, PMTT2, and PMTT5, respectively.

Table S1. Tukey simultaneous test for differences of means (confidence level = 0.95*)

Difference of Levels	Difference of Means	T-Value	Adjusted p-Value
2 - 1	-0.0362	-1.95	0.207
3 - 1	-0.1241	-6.69	< 0.001
4 - 1	-0.1995	-10.76	< 0.001
3 - 2	-0.0879	-4.74	< 0.001
4 - 2	-0.1633	-8.81	< 0.001
4 - 3	-0.0754	-4.07	< 0.001

**Individual confidence level = 98.94%*

S4. Model bacteria information

Escherichia coli CECT 101 is a rod-shaped, facultative anaerobic, mesophilic, gram-negative bacterium that was isolated from an unidentified source. *Escherichia coli* CECT 434 is also a rod-shaped, mesophilic, gram-negative bacterium; however, unlike the preceding strain, it can form biofilms. This strain is a pathogen that is resistant to several antibiotics and was clinically isolated from a human source [6]. *E. coli* species can be found in soil, water, plants, insects, birds, and mammals. It has been frequently detected in the lower intestines of warm-blooded species, including humans. It is the most researched prokaryotic organism and includes a diverse range of strains, both pathogenic and non-pathogenic [7]. Initially, *E. coli* was identified as an intestinal pathogen and a major cause of foodborne disease. Some strains can readily form biofilm on the surface of living or non-living carriers, which can lead to cross-contamination. The bacterial resistance to disinfectants increases considerably once the microbial biofilm is formed [8].

Ligilactobacillus salivarius CECT 4063 is a mesophilic, gram-positive, facultative anaerobic, rod-shaped bacterium that was isolated from human saliva [6]. Originally called *Lactobacillus salivarius*, the species *Ligilactobacillus salivarius* was renamed in March 2020 in accordance with a revised taxonomic classification by the International Committee on Systematics of Prokaryotes (ICSP) [9-10]. *L. salivarius* is a homofermentative bacterium that generates lactic acid through a carbohydrate metabolism. It is a lactic acid bacterium that may be found naturally in the human digestive tract, milk, and oral cavities, among other areas [11]. *L. salivarius* has been detected on the surface of human teeth, saliva, and tongue [12]. This bacterium has also been reported in the microbiota of the digestive tracts of chickens, pigs, ducks, and cattle [13]. *L. salivarius* is also widely recognized for its probiotic capabilities [14-15].

Streptococcus mutans CECT 479 is a microaerophilic, mesophilic, cocciform, gram-positive bacterium isolated from carious dentin [6]. *S. mutans* species live mostly in the mouth, throat, and intestines [16]. Although *S. mutans* is mostly recognized for its role in the development of dental caries, it has also occasionally been linked to subacute infective endocarditis [7]. *S. mutans* is normally a commensal microorganism present in the human oral cavity [14]. However, due to many variables, including changes in the environment and the availability of fermentable carbohydrates, it can adapt and acquire new physiological and metabolic characteristics that affect the homeostasis of the dental biofilm, colonize the tooth surface, and damage the hard tooth structure. In addition, *S. mutans* is one of the acid-producing bacteria that causes metabolic imbalances in dental biofilms (composed of various microorganisms), leading to the development of dental caries [17]. *S. mutans* is widely known for its biofilm-dependent lifestyle. Its natural habitat is the human mouth, particularly dental plaque on tooth surfaces [12].

Streptococcus sanguinis CECT 480 is a cocciform, microaerophilic, facultative anaerobic, mesophilic, gram-positive bacterium isolated from human subacute bacterial endocarditis [6,18]. The original designation of *Streptococcus sanguinis* was *Streptococcus sanguis*, meaning blood in Latin, because the only source used in the initial publication was blood from patients with endocarditis. Subsequently, for grammatical reasons, it was changed to *S. sanguinis* [18-19]. This species has only been isolated from humans and is not only one of the most common and prolific inhabitants of the tooth surface but has also developed mechanisms to protect the host from damage caused by its presence and proliferation [19]. It is one of the first to colonize the tooth surface and is frequently associated with good oral health. For example, *Streptococcus sanguinis* has been demonstrated to inhibit the growth of *S. mutans* by producing H_2O_2 [7]. These features make it a commensal bacterium abundantly dispersed in the oral cavity [20]. *S. sanguinis* is found on tooth surfaces (the preferred site of colonization), on oral mucosal surfaces, and in human saliva. It predominates in both supragingival

and subgingival plaques [18]. *S. sanguinis* has been suggested to be the agent responsible for several extraoral diseases. The most significant association is with infective endocarditis, an uncommon but potentially lethal condition. In rare circumstances, this species has caused meningitis and severe bacteremia [19].

S5. McFarland calibration curve

Standards were prepared by mixing different proportions of barium chloride solution and sulfuric acid solution. Absorbance measurements of the standards were performed with a Biochrom EZ Read 400 microplate reader at a wavelength of 620 nm. 100 µL of each standard was deposited in three different wells of a 96-well ELISA plate to perform the curve with the mean value. The basis for preparing this calibration curve is that a 0.5 McFarland standard is prepared by mixing 0.05 mL of 1.175% w/v barium chloride dihydrate (1% w/v barium chloride) with 9.95 mL of 1% v/v sulfuric acid, corresponding approximately to a cell density of 1.5×10^8 CFU/mL. The other standards were prepared by proportionally increasing the amount of barium chloride solution [21-22]. Figure S1 shows the calibration curve obtained. Five points were measured, each in triplicate. The linear regression coefficient obtained of $R^2 = 0.9921$ is sufficient for both the precision offered by this approach and the required accuracy of the experiments conducted in this study.

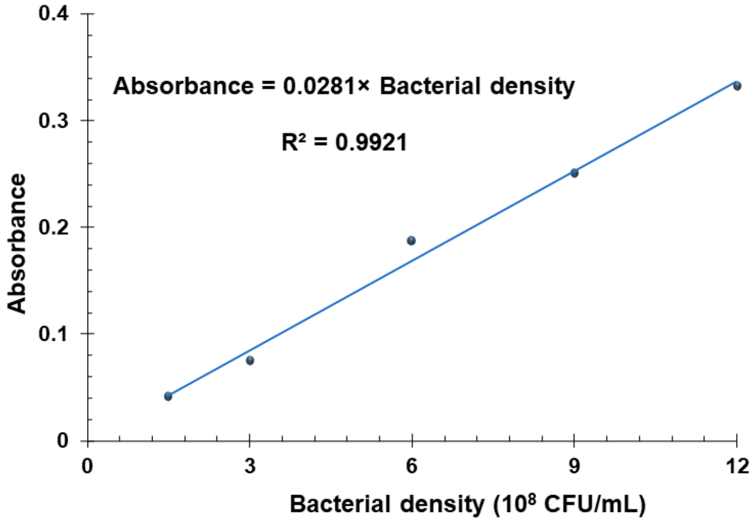

Figure S4. McFarland calibration curve

S6. Bacterial detection experiment replicating environmental conditions

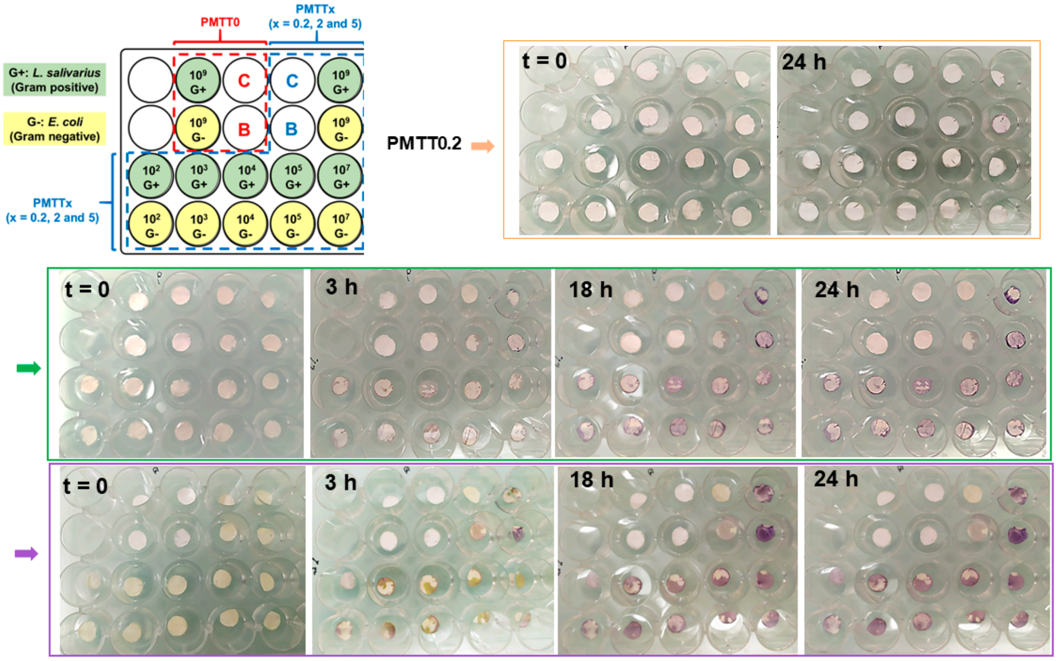

Figure S5. Photographic comparison, without cuts, of the bacterial detection limits of the MTT/PADAS mats when incubated under environmental conditions at different time points. In all cases, "C" stands for control (samples that were not immersed in sterile LB broth or in the bacterial suspension), while B stands for blank (samples submerged in clean LB broth)

References

1. Paredes, N.; Rodriguez-Galán, A.; Puiggalí, C.; Peraire, C. Studies on the Biodegradation and Biocompatibility of a New Poly(Ester Amide) Derived from L-Alanine. J. Appl. Polym. Sci. 1998, 69, 1537–1549.
2. Del Valle, L.J.; Roa, M.; Díaz, A.; Casas, M.T.; Puiggalí, J.; Rodríguez-Galán, A. Electrospun Nanofibers of a Degradable Poly(Ester Amide). Scaffolds Loaded with Antimicrobial Agents. J. Polym. Res. 2012, 19, 9792. https://doi.org/10.1007/s10965-011-9792-2.
3. Rodríguez-Galán, A.; Pelfort, M.; Aceituno, J.E.; Puiggalí, J. Comparative Studies on the Degradability of Poly(Ester Amide)s Derived from L- and L,D-Alanine. J. Appl. Polym. Sci. 1999, 74, 2312–2320. https://doi.org/10.1002/(SICI)1097-4628(19991128)74:9<2312::AID-APP21>3.0.CO;2-0.
4. Valenti, S.; Arioli, M.; Jamett, A.; Tamarit, J.L.; Puiggalí, J.; Macovez, R. Amorphous Solid Dispersions of Curcumin in a Poly(Ester Amide): Antiplasticizing Effect on the Glass Transition and Macromolecular Relaxation Dynamics, and Controlled Release. Int. J. Pharm. 2023, 644, 123333. https://doi.org/10.1016/j.ijpharm.2023.123333.
5. Van Krevelen, D.W. Properties of Polymers, 4th ed; Te Nijenhuis, K., Ed.; Elsevier: Amsterdam, The Netherlands, 2009; ISBN 978-0-08-054819-7.
6. Reimer, L.C.; Sardà Carbasse, J.; Koblitz, J.; Ebeling, C.; Podstawka, A.; Overmann, J. BacDive in 2022: The Knowledge Base for Standardized Bacterial and Archaeal Data. Nucleic Acids Res. 2022, 50, D741–D746. https://doi.org/10.1093/nar/gkab961.

7. Lemos, J.A.; Quivey, R.G.; Koo, H.; Abranches, J. Streptococcus Mutans: A New Gram-Positive Paradigm? Microbiology 2013, 159, 436–445. https://doi.org/10.1099/mic.0.066134-0.

8. Zhou, F.; Wang, D.; Hu, J.; Zhang, Y.; Tan, B.K.; Lin, S. Control Measurements of Escherichia Coli Biofilm: A Review. Foods 2022, 11, 2469. https://doi.org/10.3390/foods11162469.

9. Zheng, J.; Wittouck, S.; Salvetti, E.; Franz, C.M.A.P.; Harris, H.M.B.; Mattarelli, P.; O'toole, P.W.; Pot, B.; Vandamme, P.; Walter, J.; et al. A Taxonomic Note on the Genus Lactobacillus: Description of 23 Novel Genera, Emended Description of the Genus Lactobacillus Beijerinck 1901, and Union of Lactobacillaceae and Leuconos-tocaceae. Int. J. Syst. Evol. Microbiol. 2020, 70, 2782–2858. https://doi.org/10.1099/ijsem.0.004107.

10. Dec, M.; Stępień-Pyśniak, D.; Puchalski, A.; Hauschild, T.; Pietras-Ożga, D.; Ignaciuk, S.; Urban-Chmiel, R. Biodiversity of Ligilactobacillus Salivarius Strains from Poultry and Domestic Pigeons. Animals 2021, 11, 972. https://doi.org/10.3390/ani11040972.

11. Guerrero Sanchez, M.; Passot, S.; Campoy, S.; Olivares, M.; Fonseca, F. Ligilactobacillus Salivarius Functionalities, Applications, and Manufacturing Challenges. Appl. Microbiol. Biotechnol. 2022, 106, 57–80.

12. Wu, C.C.; Lin, C.T.; Wu, C.Y.; Peng, W.S.; Lee, M.J.; Tsai, Y.C. Inhibitory Effect of Lactobacillus Salivarius on Streptococcus Mutans Biofilm Formation. Mol. Oral Microbiol. 2015, 30, 16–26. https://doi.org/10.1111/omi.12063.

13. Rondón, A.J.; Rodríguez, M.; Beruvides, G.A. Probiotic Potential of Lactobacillus Salivarius in Animals of Zoo-technical Interest Potencial Probiótico de Lactobacillus Salivarius. Cuba. J. Agric. Sci. 2020, 54, 1–13.

14. Sañudo, A.I.; Luque, R.; Díaz-Ropero, M.P.; Fonollá, J.; Bañuelos, Ó. In Vitro and in Vivo AntiMicrobial Activity Evaluation of Inactivated Cells of Lactobacillus Salivarius CECT 5713 against Streptococcus Mutans. Arch. Oral Biol. 2017, 84, 58–63. https://doi.org/10.1016/j.archoralbio.2017.09.014.

15. Nishihara, T.; Suzuki, N.; Yoneda, M.; Hirofuji, T. Effects of Lactobacillus Salivarius-Containing Tablets on Caries Risk Factors: A Randomized Open-Label Clinical Trial. BMC Oral Health 2014, 14. https://doi.org/10.1186/1472-6831-14-110.

16. Forssten, S.D.; Björklund, M.; Ouwehand, A.C. Streptococcus Mutans, Caries and Simulation Models. Nutrients 2010, 2, 290–298.

17. Bedoya-Correa, C.M.; Rincón Rodríguez, R.J.; Parada-Sanchez, M.T. Genomic and Phenotypic Diversity of Streptococcus Mutans. J. Oral Biosci. 2019, 61, 22–31.

18. Zhu, B.; Macleod, L.C.; Kitten, T.; Xu, P. Streptococcus Sanguinis Biofilm Formation & Interaction with Oral Pathogens. Future Microbiol. 2018, 13, 915–932.

19. Kreth, J.; Giacaman, R.A.; Raghavan, R.; Merritt, J. The Road Less Traveled – Defining Molecular Commensalism with Streptococcus Sanguinis. Mol. Oral Microbiol. 2017, 32, 181–196.

20. Pauter-Iwicka, K.; Railean, V.; Złoch, M.; Pomastowski, P.; Szultka-Młyńska, M.; Błońska, D.; Kupczyk, W.; Buszewski, B. Characterization of the Salivary Microbiome before and after Antibiotic Therapy via Separation Technique. Appl. Microbiol. Biotechnol. 2023, 107, 2515–2531. https://doi.org/10.1007/s00253-023-12371-0.

21. Cockerill, F.R.; Wikler, M.A.; Alder, J.; Dudley, M.N.; Eliopoulos, G.M.; Ferraro, M.J.; Hardy, D.J.; Hecht, D.W.; Hindler, J.A.; Patel, J.B.; et al. M07-A9: Methods for Dilution Antimicrobial Susceptibility Tests for Bacteria That Grow Aerobically; Approved Standard, 9th ed.; Clinical and Laboratory Standards Institute: Wayne, NY, USA, 2012; Volume 32, ISBN 1-56238-784-7.

22. McFarland, J. Nephelometer: An Instrument for Estimating the Number of Bacteria in Suspensions Used for Calculating the Opsonic Index and for Vaccines. J. Am. Med. Assoc. 1907, 14, 1176–1178. https://doi.org/10.1001/jama.1907.25320140022001f.
